# Unilateral Convulsions and Hemiplegia with Exudative Erythema

**Published:** 1905-07-08

**Authors:** 


### UNILATERAL CONVULSIONS AND HEMIPLEGIA WITH EXUDATIVE ERYTHEMA.

Dr. T. K. Monro[1] publishes two examples of the association of these symptoms in young patients. The first patient was a boy of 12 years, who, on June 12th, 1903, began to suffer from pain and stiffness in his joints. Subsequently, vomiting and diarrhœa became troublesome, and continued for a fortnight. At this date convulsions commenced. They were confined to the right side, and affected the right face and limbs simultaneously. At first attacks were frequent, but afterwards gradually became less often, and at the end of a fortnight ceased altogether. Patient during this time never spoke, and for some days was apparently unconscious; the evacuations were passed into the bed, a bed-sore developed, and linear atrophy of the skin was observed on the knees and thigh. After some weeks speech began to return, but still shows very marked defect. Similarly some weakness of the right face and right upper limb remains. So far, therefore, the case accommodates itself to a well-recognised form of hemiplegia in children in which the paresis follows one or more convulsive seizures, and the lesion is either a vascular obstruction or an inflammation involving the motor region of the cortex. The chief interest of the case is presented as a development of erythema nodosum together with marked pyrexia some six months after the occurrence of paralysis. The erythema was not accompanied by arthritis, though in previous years the boy had had an attack of sub-acute rheumatism. It cannot be said that such an occurrence does much to throw light on the exact pathology of these obscure cases of hemiplegia in childhood. The event can be readily harmonised either with the theory of thrombosis or with that of inflammation (polio-encephalitis). Further, as an interval of something like six months separated the occurrence of the paralysis from the development of the erythema, it is at least possible, to put it mildly, that the two occurrences were the expression not of the same but of different causes.

The second patient was a girl of 18 years who was found unconscious and "kicking." Examination showed the presence of right hemiplegia, and right-sided convulsions were observed beginning in the arm and spreading first to the leg and afterwards to the face, this series of events being frequently repeated during a period of three days. The seizures were attended by loss of consciousness, but in the intervals patient was conscious and able to speak. The right plantar response was of the extensor type. In the course of a week signs of returning power in the right limbs could be appreciated, but there were evidences of paresis of both external recti muscles, and headache and vomiting were additional symptoms. Further, double optic neuritis had now developed. In a little time, however, these symptoms all subsided, and in a few weeks the patient left the hospital quite well except for a slight degree of weakness and spasticity in the right leg. The sex and age of the patient, and certain facts in the history, such for example as amenorrhœa, seem to be sufficient to indicate that this case belongs to a well-recognised group in which acute and severe cerebral symptoms develop in patients the subject of chlorosis. The one lesion which has been discovered in these cases is intracranial thrombosis. The presence of the paresis of the sixth nerves is interesting, but this observation has been made in several previous cases. Thus, so far, there seems nothing in the case to differentiate it from the cases of hemiplegia and other cerebral events which occasionally occur in chlorosis. The special point on which Dr. Munro places stress is the development of a measly eruption on the limbs and trunk, with more or less hyperæmia of the trunk. The patient had previously suffered from rheumatic fever, with "red blotches" on the skin, and she presented signs of local asphyxia in each of the four extremities. All this hardly helps forward any of the recognised theories regarding the exact cause of the cerebral disturbances of chlorosis. It is well to have a record of these cases, but in view of all the circumstances the erythema can hardly be said to lend either one case or the other so distinctive a character as to call for separate classification.

[1] Brit. Med. Jour., May 27, 1905.

